# Challenges, Strategies, and Perspectives for Reference-Independent Longitudinal Multi-Omic Microbiome Studies

**DOI:** 10.3389/fgene.2021.666244

**Published:** 2021-06-14

**Authors:** Susana Martínez Arbas, Susheel Bhanu Busi, Pedro Queirós, Laura de Nies, Malte Herold, Patrick May, Paul Wilmes, Emilie E. L. Muller, Shaman Narayanasamy

**Affiliations:** ^1^Luxembourg Centre for Systems Biomedicine, University of Luxembourg, Esch-sur-Alzette, Luxembourg; ^2^Department of Environmental Research and Innovation, Luxembourg Institute of Science and Technology, Belvaux, Luxembourg; ^3^Department of Life Sciences and Medicine, Faculty of Science, Technology and Medicine, University of Luxembourg, Esch-sur-Alzette, Luxembourg; ^4^Université de Strasbourg, UMR 7156 CNRS, Génétique Moléculaire, Génomique, Microbiologie, Strasbourg, France

**Keywords:** microbiome, metatranscriptomics, metaproteomics, time-series, metagenomics, metabolomics, *de novo assembly*

## Abstract

In recent years, multi-omic studies have enabled resolving community structure and interrogating community function of microbial communities. Simultaneous generation of metagenomic, metatranscriptomic, metaproteomic, and (meta) metabolomic data is more feasible than ever before, thus enabling in-depth assessment of community structure, function, and phenotype, thus resulting in a multitude of multi-omic microbiome datasets and the development of innovative methods to integrate and interrogate those multi-omic datasets. Specifically, the application of reference-independent approaches provides opportunities in identifying novel organisms and functions. At present, most of these large-scale multi-omic datasets stem from spatial sampling (e.g., water/soil microbiomes at several depths, microbiomes in/on different parts of the human anatomy) or case-control studies (e.g., cohorts of human microbiomes). We believe that longitudinal multi-omic microbiome datasets are the logical next step in microbiome studies due to their characteristic advantages in providing a better understanding of community dynamics, including: observation of trends, inference of causality, and ultimately, prediction of community behavior. Furthermore, the acquisition of complementary host-derived omics, environmental measurements, and suitable metadata will further enhance the aforementioned advantages of longitudinal data, which will serve as the basis to resolve drivers of community structure and function to understand the biotic and abiotic factors governing communities and specific populations. Carefully setup future experiments hold great potential to further unveil ecological mechanisms to evolution, microbe-microbe interactions, or microbe-host interactions. In this article, we discuss the challenges, emerging strategies, and best-practices applicable to longitudinal microbiome studies ranging from sampling, biomolecular extraction, systematic multi-omic measurements, reference-independent data integration, modeling, and validation.

## Introduction

Advances in the study of microbial communities have highlighted their important role in natural processes, including those considered as ecosystem services for humankind (Bodelier, 2011). Complex dynamics in microbiomes at the level of composition and structure, as well as function (Heintz-Buschart and Wilmes, 2018) stem from constant adaptation of a given community toward fluctuations of abiotic and biotic factors. However, the fate of these microbial consortia in the face of perturbations is often not understood nor predictable (Muller, 2019). Longitudinal approaches are necessary to understand microbial community dynamics, as they may offer valuable insights into temporal trends and consequences of environmental forcings, when used in tandem with host-derived (Heintz-Buschart et al., 2016; Lloyd-Price et al., 2019; Mars et al., 2020) or environmental (Law et al., 2016; Herold et al., 2020) data. Longitudinal studies can be conducted using diachronic or synchronic approaches (Costa Junior et al., 2013). Herein, we discuss the capacity of longitudinal diachronic approaches as a critical tool toward studying microbial communities. We will further focus on multi-omics longitudinal studies, which leverage the power of the entire high-throughput meta-omic spectrum, namely meta-genomics (MG), -transcriptomics (MT), -proteomics (MP), and -metabolomics (MM), as they are now more feasible and affordable than ever before (Narayanasamy et al., 2015).

Overall, longitudinal multi-omics will enhance our understanding of microbial community dynamics, which could potentially bring about positive outcomes in biomedicine, biotechnology, and for the environment. However, various aspects must be considered when conducting longitudinal multi-omic microbiome studies, ranging from experimental design, bioinformatic processing, modeling, and validation. In this article, we explore challenges, considerations, and potential solutions for such studies, based on recent advances and reports (Law et al., 2016; Lloyd-Price et al., 2019; Herold et al., 2020; Martínez Arbas et al., 2021), which are applicable to both microbe-centric (e.g., soil, water) or host-centric (e.g., human gut) systems. Finally, although this article focuses on specifically longitudinal multi-omic microbiome studies, the content is generally applicable to any large-scale microbiome studies.

## Multi-Omic Considerations and Experimental Design for Longitudinal Studies

Integration of multi-omic microbiome datasets has been routinely performed, with notable instances, including studies on type-1 diabetes (Heintz-Buschart et al., 2016), cancer (Kaysen et al., 2017), healthy human gut (Tanca et al., 2017), Crohn’s disease (Erickson et al., 2012), and activated sludge (Muller et al., 2014; Roume et al., 2015; Yu et al., 2019). These studies clearly demonstrate the maturity of the current microbiome multi-omics toolbox. Despite this, and to the best of our knowledge, equivalent multi-omic surveys based on extensive longitudinal microbiome sampling remain rather limited. Table 1 lists several relevant studies of longitudinal (at least six timepoints) and multi-omic (at least two omic levels, excluding 16S amplicon sequencing) microbiome datasets.

**Table 1:** Longitudinal multi-omic microbiome datasets and studies.

System	Sample type	Duration^∗^	Frequency^∗^	Total of samples	MG	MT	MP	MM	Complementary data	Studies
Human gut microbiome	Stool samples from 132 humans; healthy or with Crohn’s disease or ulcerative colitis	1 year	Bi-weekly	2,965	x	x	x	x	Host genomics, transcriptomics bisulfite sequencing, serologic profiles, diet surveys, and fecal calprotectin	Lloyd-Price et al., 2019
										Ruiz-Perez et al., 2021
	Stool samples of 77 individuals	6 months	Monthly	474	x			x	Host transcriptome, metabolome, cytokines, methylome, dietary survey, and physiology	Blasche et al., 2021
Activated sludge	Floating sludge islets from a single anoxic tank	1.5 year	Weekly	53	x	x	x	x	Temperature, pH, oxygen concentration, conductivity, inflow, nitrate concentration, and extracellular metabolites	Herold et al., 2020
										Martínez Arbas et al., 2021
	Full- and lab-scale activated sludge	2.5 months	Weekly	10	x	x			Temperature, pH, redox potential and dissolved oxygen	Law et al., 2016

Longitudinal multi-omic data must be of least six timepoints and at least two meta-omic readouts excluding 16S amplicon sequencing. Omics data derived from host(s) are considered separate from the microbial meta-omic spectra.

∗ Approximate values.

**Figure 1:**
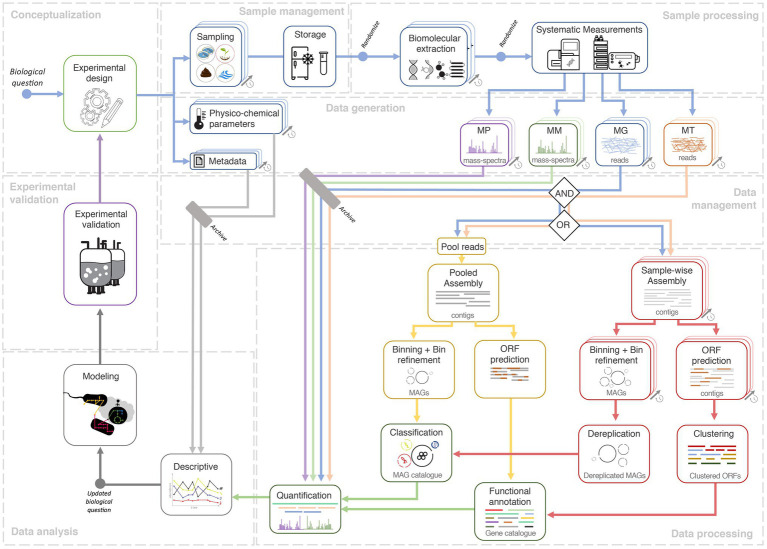
Systems ecology workflow for longitudinal multi-omic microbiome studies. A study conceptualized *via* an experimental design phase and an initial biological question which is then followed by sample collection, sample management, and systematic high-throughput measurements. The next-generation sequencing (NGS) data could either undergo aggregated processing (yellow track) involving a pooled *de novo* assembly of NGS reads from all longitudinal samples, to eventually yield a metagenome assembled genome (MAG) and/or gene catalogue *via* binning and gene calling, respectively. In the dereplication approach (red track), data from each sample are first processed in a sample-wise manner, namely the steps of *de novo* assembly, binning, and gene calling. The resulting MAGs and predicted ORFs are then merged through a process called dereplication which generates the catalogue. The availability of a catalogue allows quantification whereby the output could be used for descriptive analyses which could potentially lead to updated or entirely novel biological questions. Quantified values, combined with descriptive analyses, could then be used within dynamic or metabolic models (gray track). Validation of models could lead to further *in situ* longitudinal experimental designs. Finally, all data (raw input, output, metadata) and code (not depicted) should be archived under a data and code management strategy. Free icons were used from https://www.flaticon.com (creators: Freepik, Gregor Cresnar, Freepik, and Smashicons).

The famous adage “*absence of evidence is not evidence of absence*” (Altman and Bland, 1995) could likely be a prelude to most microbiome studies. Hence, we discuss these studies in the context of reference-independent bioinformatics approaches, centered around *de novo* assemblies of sequencing data (MG and MT), subsequently complemented by additional omics (MP and MM, depending on their availability; Figure 1). Reference-independent approaches offer asymmetric advantages and opportunities in discovering novel microbial taxa and/or functionalities (Celaj et al., 2014; Narayanasamy et al., 2015; Lapidus and Korobeynikov, 2021), compared to reference-dependent methodologies (Sunagawa et al., 2013; Treangen et al., 2013). Moreover, the integration of multi-omics has been shown to yield superior output compared to single omic studies. For instance, the co-assembly of MG and MT sequencing reads was shown to improve the quality of assembled contigs (Narayanasamy et al., 2016), which in turn improves taxonomic annotation, gene calling/annotation, binning, metabolic pathway (re) construction (Muller et al., 2018; Zhou et al., 2020; Zimmermann et al., 2021), and quantification of features, e.g., taxa/genes (Narayanasamy et al., 2016). Similarly, MP spectra searches are more effective when performed against gene databases derived from MG assemblies of the same sample/environment, compared to generic databases, thus improving the recruitment of measured peptides (Tanca et al., 2016; Heyer et al., 2017; Timmins-Schiffman et al., 2017). Moreover, such a reference-independent approach may be necessary for microbial communities that are not well characterized and lack extensive unified genome or gene catalogues, such as those available for the human gut microbiome (Li et al., 2014; Almeida et al., 2021). However, most microbial communities are heterogeneous, which further complicates downstream multi-omic data processing, integration, curation, transformation, and modeling (Jiang et al., 2019). Therefore, the adherence toward standards and best-practices, spanning from sampling to data analyses is important to the outcome of a project. Accordingly, Figure 1 illustrates the potential lifecycle of a longitudinal multi-omic microbiome study.

Longitudinal multi-omic studies require systematic and thorough study designs that consider sampling parameters (Gerber, 2014; Cao et al., 2017; Liang et al., 2020), metadata, and complementary measurements, such as physico-chemical parameters or questionnaires (Kumar et al., 2014), all of which affect downstream analyses. Sampling parameters, such as duration and frequency, are dictated by the inherent properties of a given microbial system. For instance, the sampling duration when studying gut microbiome development of neonates could span from birth until a “mature” gut microbiome composition is achieved (Stewart et al., 2018), which may vary from subject to subject. Naturally-occurring microbial systems that are exposed to the environment may exhibit annual cyclical behavior based on seasonality and, therefore, could be sampled for at least one complete season-to-season cycle (Johnston et al., 2019). Sampling frequency may be determined by the dynamics and/or generational-timescale of a given system. For instance, the human gut microbiome is known to exhibit daily fluctuations, and therefore could be sampled on a daily basis within a given temporal study (David et al., 2014), while activated sludge systems are known to exhibit (approximately) weekly doubling periods and thus could be sampled on a weekly basis (Herold et al., 2020; Martínez Arbas et al., 2021). Based on the recommendations of Sefer et al. (2016), if biological replicates are either not feasible (i.e., *n* = 1) or limited (i.e., low *n*) (Herold et al., 2020), one should ideally opt for higher frequency (dense) longitudinal sampling, and less dense sampling if biological replicates were available (i.e., high *n*), e.g., a cohort of patients (Lloyd-Price et al., 2019). Equidistant sampling is required by many downstream mathematical frameworks, such as cross-correlation or local similarity analysis (Faust et al., 2015), and thus should be strived for, as much as possible. However, the datasets listed in Table 1, albeit extensive and resource intensive, are not perfectly equidistant, further highlighting the practical challenges for longitudinal sampling *in situ*, including, but not limited to, accessibility, consistent biomass availability, and cost.

## Sample, Data and Code Management

It is crucial to limit potential biases linked to longitudinal data, e.g., in extended time-series; samples are stored for long periods, while multiple personnel may be involved in sample collection, handling, storage, and documentation. Hence, clear guidelines and standardization must be established, as they are key factors that potentially affect downstream processes and overall outcome (Blekhman et al., 2016; Schoenenberger et al., 2016).

Biomolecular extraction from a single sample is ideal over multiple extractions from subsamples (Roume et al., 2013a). Advantageously, commercial kits for concomitant extraction of multiple biomolecules are available, including reports proposing adapted methods for extracting various biomolecules, such as DNA, total RNA, small RNA, protein, and metabolites (Peña-Llopis and Brugarolas, 2013; Roume et al., 2013b; Thorn et al., 2019). The availability of sufficient biomass (Eisenhofer et al., 2019) lysis-, homogenization-(Machiels et al., 2000; Santiago et al., 2014; Fiedorová et al., 2019) and preservation- (Borén, 2015; Hickl et al., 2019) methods are key factors that determine effectiveness to comprehensively recover all intracellular and/or extracellular biomolecules. Next, biomolecular extraction should be automated, whenever possible. While evaluations have shown that it may not necessarily provide better quality results compared to a human operator (Phillips et al., 2012), the output is more consistent (Fidler et al., 2020). In the same vein, omic readouts should also be generated on a single platform (s) as unique batches to ensure consistent output quality.

Batch effects are often overlooked in omic studies (de Goffau et al., 2021), but can be minimized during stages of sample processing by including randomization, sample tracking, and extensive documentation (Leek et al., 2010). Sample randomization implemented within batches of biomolecular extraction and high-throughput measurements could help discriminate batch effects and temporal variation, i.e., different sets of randomly selected samples from different timepoints could be treated together at each different step (Oh et al., 2019). Additionally, batch effects could be mitigated using downstream analytical (Wang and Cao, 2019) and computational methods (Gibbons et al., 2018; McLaren et al., 2019).

A potential effective experimental measure for minimizing and elucidating batch effects is the inclusion of mock/control samples during both the extraction and high-throughput measurements (Bokulich et al., 2016; Hornung et al., 2019; ATCC Mock Microbial Communities, 2020). Samples with low biomass, e.g., from neonates, glacier-streams, or acid-mine drainage, should include extraction blanks as negative controls, which are extremely valuable to discriminate contaminants arising from kits and reagents (Salter et al., 2014; Heintz-Buschart et al., 2018; Wampach et al., 2018; Weyrich et al., 2019). Furthermore, spike-ins could be helpful for downstream quantification (Zinter et al., 2019). Importantly, replicates can be used within downstream statistical frameworks (Sokal, 1995; Anderson, 2017; Kuznetsova et al., 2017; Mallick et al., 2021) to understand both within- and between-sample heterogeneity, thereby minimizing mischaracterisation of contaminants or findings driven by batch effects (de Goffau et al., 2021).

Longitudinal and multi-omic studies yield large datasets, where data processing and analyses are typically time and resource intensive. These rich datasets may be reused to study multiple aspects of a given microbial system (Table 1). Therefore, equal emphasis should be placed on designing bioinformatic workflows and code/data management strategies to improve reproducibility and transparency. For example, peer-review journals have begun mandating “data availability” sections and links to code repositories in adherence to project/coding best practices and standards (Sandve et al., 2013; Bokulich et al., 2020), further improving posterior data integration and analysis in the short-term, while improving scaling-up and knowledge transfer in the long run (Shahin et al., 2017; Wilson et al., 2017). In addition, format-free archival repositories, such as Zenodo could be used for non-standard data types,^1^ for instance simulated raw data, physico-chemical measurements, intermediate data, large tables, and archived Github repositories. Despite this, reports indicate that 26% of bioinformatics tools are no longer available (Mangul et al., 2019), while gaps in available raw data (Jurburg et al., 2020) and metadata (Schriml et al., 2020) still exist.

## Construction of Longitudinal Gene and Genome Reference Catalogues

Microbiomes may be studied from a gene-centric perspective (Roume et al., 2015), which requires read or contig-level taxonomic classification (Segata et al., 2012; Wood and Salzberg, 2014), ORF prediction (Hyatt et al., 2010; Rho et al., 2010), and gene annotation (Seemann, 2014; Buchfink et al., 2015; Franzosa et al., 2018; Queirós et al., 2020). Metagenome assembled genomes (MAGs) provide genomic context and can be obtained through binning (Chen et al., 2020; Yue et al., 2020) followed by taxonomic classification (Bremges et al., 2020; Chaumeil et al., 2020) and functional annotation. In that regard, several tools exist that improve the binning process by automating the selection of highest-quality MAGs (bins) and/or performing MAG refinement (Broeksema et al., 2017; Sieber et al., 2018; Uritskiy et al., 2018). These tools enable ensemble binning approaches, balancing out the strengths and weaknesses of different binning methods (Chen et al., 2020; Yue et al., 2020).

Features (i.e., taxa or genes) appear in varying quantities, in different timepoints of longitudinal meta-omic studies. It is challenging to link and track features from one timepoint to another without any given point of reference. Therefore, the construction of what we term as “representative longitudinal catalogues” (hereafter referred to as catalogues) of MAGs/genes, provides a non-redundant representative base to link features from the different longitudinal samples (Herold et al., 2020; Martínez Arbas et al., 2021). The outcome of any downstream analysis is highly reliant on the quality of the MAGs and genes within a catalogue, which further depends on the quality of large-scale bioinformatic processing (e.g., *de novo* assembly and binning). Figure 1 illustrates two methods of constructing such catalogues, which are through aggregated processing of data from all samples or through de-replicating the output from individually processed sample data (i.e., sample-wise processing). A third alternative to these methods could be the representation of non-redundant genes in pangenomes from MAGs annotated at the species-level (Tettelin et al., 2005; Delmont and Eren, 2018), collected across all timepoints. This allows for identifying any varying patterns especially in the context of environmental factors and phylogenetic constraints influencing gene acquisition and/or genome-streamlining (Tettelin et al., 2005). Given that others have highlighted the catalogue building methodologies (Qin et al., 2010; Nayfach et al., 2020; Almeida et al., 2021); here, we elaborate methods discussed above in the context of both gene- and MAG-centric strategies.

The general advantage of the aggregated processing approach is simplicity, whereby a single run is required for all the large-scale bioinformatic processing steps (Figure 1). Moreover, pooled assemblies have been shown to be effective (Magasin and Gerloff, 2015), especially in the advent of highly efficient *de novo* assemblers (Li et al., 2016) and digital normalization (Brown et al., 2012). However, pooling reads from a large number of samples increases the complexity of the *de novo* assembly process, especially for complex communities. It also requires substantial computational resources, while potentially resulting in lower quality contigs, MAGs, and genes (Chen et al., 2020).

The dereplication method (Figure 1) is applied after independent sample-wise large-scale bioinformatic processing (Evans and Denef, 2020). Predicted ORFs could be de-replicated through clustering (Li and Godzik, 2006; Edgar, 2010; Mirdita et al., 2019), producing a gene catalogue (Li et al., 2014). On the contrary, the dereplication of MAGs is more complex, requiring several steps: binning from sample-wise *de novo* assemblies to generate MAGs, curation of high-quality MAGs (Parks et al., 2015), and dereplication of MAGs (Olm et al., 2017; Wampach et al., 2018) to select the most representative MAGs of the longitudinal data (Uritskiy et al., 2018; Chen et al., 2020). In general, dereplication methods are particularly advantageous for longitudinal microbiome studies with many deeply sequenced samples (Herold et al., 2020; Martínez Arbas et al., 2021).

Although not systematically evaluated, one caveat worth considering when constructing a catalogue based on *de novo* assemblies, binning, and dereplication is the potential loss of resolution in population-level diversity (Kashtan et al., 2014; Evans and Denef, 2020; Quince et al., 2020), which may include single nucleotide variants, copy number variants, strains, and auxiliary gene content (Evans and Denef, 2020) potentially impacting important downstream steps, such as integration of metaproteomic data (Tanca et al., 2016) or time-resolved strain tracking (Brito and Alm, 2016; Zlitni et al., 2020). To the best of our knowledge, the extent of the impact has yet to be systematically investigated. In our opinion, several strategies can be applied to overcome this issue, including the usage of a comparative genomics methodology, i.e., pangenomes (Delmont and Eren, 2018), even opt for (re) assemblies of read subsets associated to particular taxa or MAGs of interest (Albertsen et al., 2013), or the application of strain-level analysis tools (Anyansi et al., 2020).

Overall, choosing the specific methods for constructing a longitudinal catalogue depends on various factors, including the biological question, complexity of the community (van der Walt et al., 2017), number of samples, and sequencing depth. To the best of our knowledge, a comparison between an aggregated processing approach and a dereplication approach has yet to be conducted. Such a comparison would further help to inform researchers on selecting the best strategy for longitudinal analyses.

## Quantification and Normalization

Longitudinal catalogues provide compositional information of community taxa and potential functions. However, the relative quantification of community members and functionalities is key in harnessing the power of longitudinal microbiome data, as it allows the observation of community taxa/functional dynamics and could be used in downstream modeling. In that regard, quantifying MG and MT sequencing data is a standard process of aligning reads (Li and Durbin, 2009) to relevant catalogues, and then quantifying features of interest (e.g., population/gene relative genomic abundance, gene expression) based on those alignments, providing information on community structure, functional potential, and gene expression. Complementally, MP data provide functional insights, whereby several methods are available for the quantification of such data (Delogu et al., 2020; Pible et al., 2020), while identification and quantification of metabolites through MM data (Kapoore and Vaidyanathan, 2016; Mallick et al., 2019; Røst et al., 2020) provide insights on the community phenotype (s). However, *in situ* measurements of substrate uptake through labeling-based approaches (Starr et al., 2018) are challenging. Therefore, specific metabolites of interest could be indirectly linked to members of a microbial community by proportionally assigning the relative contribution of a MAG to a given (re) constructed metabolic pathway based on genomic abundance or gene/protein expression (Noecker et al., 2016; Blasche et al., 2021).

Normalization of quantified values is required to enable community structure and function comparisons between timepoint samples. The selection of normalization methods is important as it affects downstream analytical steps. There are several methods to normalize longitudinal MG and MT data, from the generation of compositional data to log-ratios and differential rankings (Chen et al., 2018; Pereira et al., 2018; Morton et al., 2019). Additionally, one should also inspect the data for potential confounding batch effects and take it into consideration when performing normalization (Gibbons et al., 2018; McLaren et al., 2019; Coenen et al., 2020). In summary, effective relative quantification and normalization will serve as a strong basis for downstream modeling approaches, and the development of robust methods for absolute quantification will be decisive in the future.

## Analysis of Community Characteristics and Dynamics

Generally, microbiome omic data are complex, as it is (i) compositional, e.g., provided as relative abundances, which require specific considerations when selecting statistical analyses (Gloor et al., 2017), (ii) highly sparse, such that the interpretation of zero-values generated from sampling, biological, or technical processes heavily affects data-derived conclusions (Silverman et al., 2020), and (iii) high dimensional, which increases modeling difficulty due to the influence of feature selection that heavily affect potential predictions (Bolón-Canedo et al., 2016). Furthermore, multi-omic studies may contain gaps within the omic spectrum, such that certain samples may not be represented within a certain omic layer (Lloyd-Price et al., 2019). Despite introducing complexity, the complementary use of different omics could improve analysis outcomes and add predictive power to models (Muller et al., 2013; Fondi and Liò, 2015). Longitudinal data introduce another layer of complexity, i.e., time dependencies, such that one timepoint is dependent on the previous timepoints, rendering conventional statistical analyses unsuitable as they assume samples to be independent (Coenen et al., 2020). This is further compounded by the fact that samples from longitudinal *in situ* studies are often low in number and non-equidistant (Park et al., 2020). Imputation may be used to supplement missing values (i.e., omic measurements or timepoints; Jiang et al., 2020).

Initial exploration of the microbiome dynamics can be assessed through ordination analyses, where high dimensional population structure data are visualized in a two-dimensional space to observe the trajectory of the samples and the behavior of the system, i.e., metastability, cycles, and alternative states (Gonze et al., 2018). Then, community member relationships may be inferred using, e.g., correlation methods (Faust et al., 2012; Friedman and Alm, 2012; Weiss et al., 2016). Unfortunately, correlations may be insufficient to assess complex community interactions, whereby the application of modeling approaches would be necessary to resolve those relationships (Fisher and Mehta, 2014; Trosvik et al., 2015; Ridenhour et al., 2017). Modeling could serve as a means of integrating several layers of omic data (Lloyd-Price et al., 2019; Ruiz-Perez et al., 2021) further elucidating microbial interplay beyond species abundances and functional potential.

Extensive literature of statistical and mathematical frameworks for multi-omic and/or longitudinal microbiome data is currently available. For instance, Noor et al. (2019) review the integration of multi-omics data from data-driven and knowledge-based perspectives. Coenen et al. (2020) discuss approaches to characterize temporal dynamics and to identify periodicity of populations and putative interactions between them, while Faust et al. (2018) propose a classification scheme for better model selection. Bodein et al. (2019) provide a multivariate framework to integrate longitudinal and multi-omics data, while Park et al. (2020) discuss the development of models and software tools for time-series metagenome and metabolome data. Overall, the application of these methodologies should be tailored toward specific hypotheses and studies, for which data exploration is essential to select modeling approaches that fit the type, quality, and quantity of the data.

More recently, the emergence of studies which track microbiome dynamics of cohorts over time, i.e., multiple individuals/sites (Carmody et al., 2019; Lloyd-Price et al., 2019; Mars et al., 2020), necessitates the ability to discriminate variation stemming from the same individual/environment compared to those from different individuals/environments. In such cases, multi-level statistical modeling (also known as mixed-effects/hierarchical models) is able to account for repeated sampling or nested variation across a sample population (Sokal, 1995; Anderson, 2017; Kuznetsova et al., 2017; Mallick et al., 2021). Most notably Lloyd-Price et al. (2019) extensively applied such methods to associate multi-omic microbiome signatures with host-derived molecular profiles in a cohort of 132 individuals. Other instances include multi-omic longitudinal studies that combine murine and human datasets to unveil the adaptation of gut microbiomes to raw and cooked food (Carmody et al., 2019) and the identification of therapeutic targets for irritable bowel syndrome (Mars et al., 2020). Finally, there are newer methodologies that apply similar/related statistical frameworks to modeling multi-omic data (Mallick et al., 2021).

The validation of the models remains one of the most challenging issues. Mathematical models combined with culture of synthetic microbial communities are commonly utilized to study mechanisms behind host-microbiome interactions (Moejes et al., 2017). It is also possible to validate interactions between microbes by, e.g., applying environmental perturbations in controlled conditions (Law et al., 2016; Herold et al., 2020). These explorations may result in a further understanding of the role of biotic and abiotic factors in shaping microbiomes, in relation to community phenotypes found in nature, biotechnological processes (Law et al., 2016; Herold et al., 2020), or host-associated microbiomes (Moejes et al., 2017; Garza et al., 2018).

## Conclusion

Longitudinal microbiome studies combined with integrated multi-omic measurements provide unprecedented opportunities to study microbial community dynamics, both structurally and functionally. In tandem with evolving high-throughput technologies, e.g., long-read sequencing (Moss et al., 2020; Wickramarachchi et al., 2020), these studies will become important tools in the exploration and potential exploitation of microbial consortia. We described strategies to mitigate the various challenges associated with such studies, encompassing study design, best practices, practical considerations, and bioinformatics processing and modeling. While longitudinal multi-omics datasets are currently scarce (Table 1), we are confident that it will increasingly become more common, similar to how we are increasingly transitioning from single omics to multi-omic (Noor et al., 2019). Longitudinal microbiome multi-omics will serve as an important tool for further improving analytical methods, which will in turn lead to relevant biomedical, biotechnological, and environmental outcomes.

## Author Contributions

SMA and SN outlined the manuscript and coordinated the writing process. LdN, SN, and SMA prepared the figure. All authors contributed to the writing, reviewing, and editing of the manuscript. All authors approved the submitted version.

### Conflict of Interest

The authors declare that the research was conducted in the absence of any commercial or financial relationships that could be construed as a potential conflict of interest.

## Footnotes

^1^https://zenodo.org
